# Chaperone Mediated Autophagy Substrates and Components in Cancer

**DOI:** 10.3389/fonc.2020.614677

**Published:** 2021-02-12

**Authors:** Javiera Rios, Alvaro Sequeida, Amelina Albornoz, Mauricio Budini

**Affiliations:** ^1^ Molecular and Cellular Pathology Laboratory, Dentistry Faculty, Institute in Dentistry Sciences, University of Chile, Santiago, Chile; ^2^ Fundación Ciencia & Vida, Santiago, Chile; ^3^ San Sebastian University, Santiago, Chile; ^4^ Autophagy Research Center (ARC), Santiago, Chile

**Keywords:** CMA, oncogenic protein, tumor suppressor, autophagy, cancer progression

## Abstract

Chaperone-mediated autophagy (CMA) represents a specific way of lysosomal protein degradation and contrary to macro and microautophagy is independent of vesicles formation. The role of CMA in different physiopathological processes has been studied for several years. In cancer, alterations of the CMA principal components, Hsc70 and Lamp2A protein and mRNA levels, have been described in malignant cells. However, changes in the expression levels of these CMA components are not always associated with changes in CMA activity and their biological significance must be carefully interpreted case by case. The objective of this review is to discuss whether altering the CMA activity, CMA substrates or CMA components is accurate to avoid cancer progression. In particular, this review will discuss about the evidences in which alterations CMA components Lamp2A and Hsc70 are associated or not with changes in CMA activity in different cancer types. This analysis will help to better understand the role of CMA activity in cancer and to elucidate whether CMA can be considered as target for therapeutics. Further, it will help to define whether the attention of the investigation should be focused on Lamp2A and Hsc70 because they can have an independent role in cancer progression beyond of their participation in altered CMA activity.

## Autophagy

**Figure 1:**
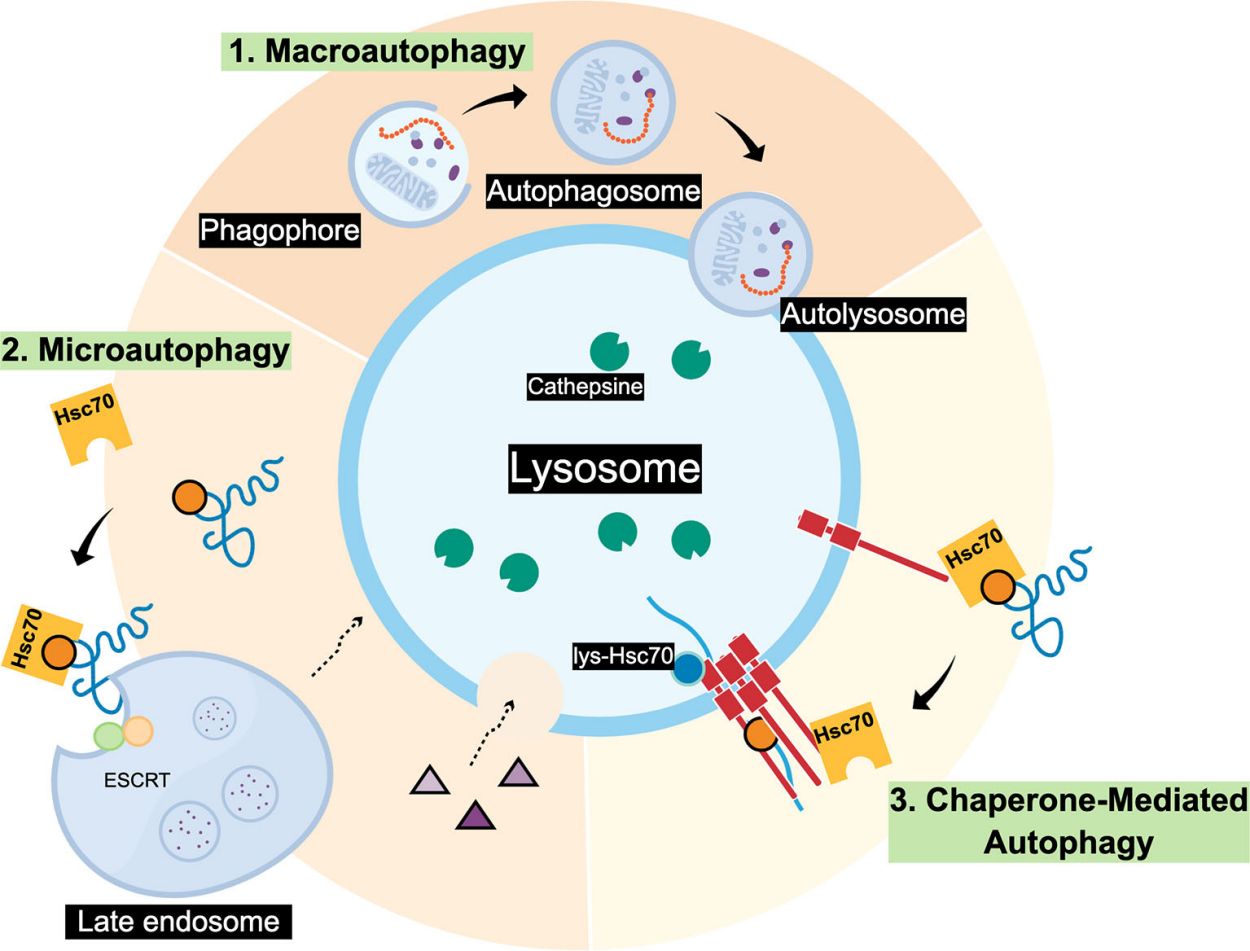
Scheme of three main autophagic pathways. 1. Macroautophagy can degrade individual proteins and complete organelles by engulfing them within an initial membrane called Phagophore, this membrane closes forming the Autophagosome vesicle. Later, the Autophagosome fuses with the lysosome membrane forming the Autolysosome where the cargo will be degraded by cathepsins and other lysosomal proteases; 2. Microautophagy, that in mammals involves the invagination of proteins (blue) that may contain a KFERQ-like motif recognized by Hsc70. Also, the ESCRT complex present in the membrane of late endosomes can form multivesicular bodies (MVBs) containing the cargo to be degraded. Later, MVBs can fuse with the lysosome. In yeast it has been observed that Microautophagy process occurs by direct invagination of the lysosomal membrane to degrade the cargo in the lumen; 3. Chaperone-mediated autophagy (CMA), necessarily requires that substrate proteins possess KFERQ-like motifs to be recognized by Hsc70. Contrary to Microautophagy, CMA also requires the Lamp2A lysosomal protein receptor located at the lysosomal membrane (red) to translocate the unfolded substrate (blue) into the lysosome lumen.

Autophagy is a lysosomal dependent cellular pathway that mediates the degradation of organelles, protein aggregates and specific proteins, and is essential for cell survival, development and homeostasis (1). There are three main routes where different cargos arrive to the lysosomes (**Figure 1**): i) Macroautophagy (MA) the cargo is trapped in double membrane vesicles known as phagosomes or autophagosomes that then fuse with the lysosome for the final cargo degradation (2); ii) Microautophagy, the lysosomes directly engulf cargo by membrane invagination (yeast) or late endosomes that form multivesicular bodies (mammals) to capture specific cytosolic components and then fuse with the lysosome for its degradation (3, 4); and (iii) Chaperone-mediated autophagy (CMA), there are no trafficking membranous vesicles involved, instead, cargo is selectively recognized by a chaperone protein and then internalized into the lysosome for its degradation (5). In the case of Microautophagy and CMA, both pathways need the interaction between the KFERQ-like motive in the protein substrate and the Hsc70 for target degradation. However, Microautophagy, does not requires Lamp2A as protein receptor (**Figure 1**) [for a compressive review see (5)].

The evidences reveal that alterations in autophagy are implicated in multiple human diseases and pathophysiological conditions like neurodegeneration (6), infection, immunity and inflammation (7), cardiovascular diseases (8), respiratory affections (9), liver diseases (10) and cancer (11). In the case of CMA, this pathway has been reported to be altered, principally, in neurodegeneration (12), metabolic disorders (12) and cancer (13). In this review, we will focus in the observations that link CMA with cancer. In particular, we will discuss the relevance that changes in CMA substrates and principal components would have in the context of different cancer types.

## Chaperone Mediated Autophagy (CMA)

The main feature of this particular lysosomal pathway is the cargo selectivity. CMA degrades mostly cytosolic proteins that are characterized by the presence of a consensus pentapeptide known as the KFERQ-like motif. This motif includes at least two positively charged residues (K, R), up to two hydrophobic residues (F), one negatively charged residue (E) and a glutamine (Q) that can be positioned at the beginning or at the end of the motif. Proteins containing the KFERQ-like motif are recognized by the Heat Shock Cognate 70 (Hsc70) protein and other co-chaperones (14) that assist in the transport to the lysosomal surface to interact with the cytosolic tail of Lysosome-associated membrane protein type 2A (Lamp2A) (5).

**Figure 2:**
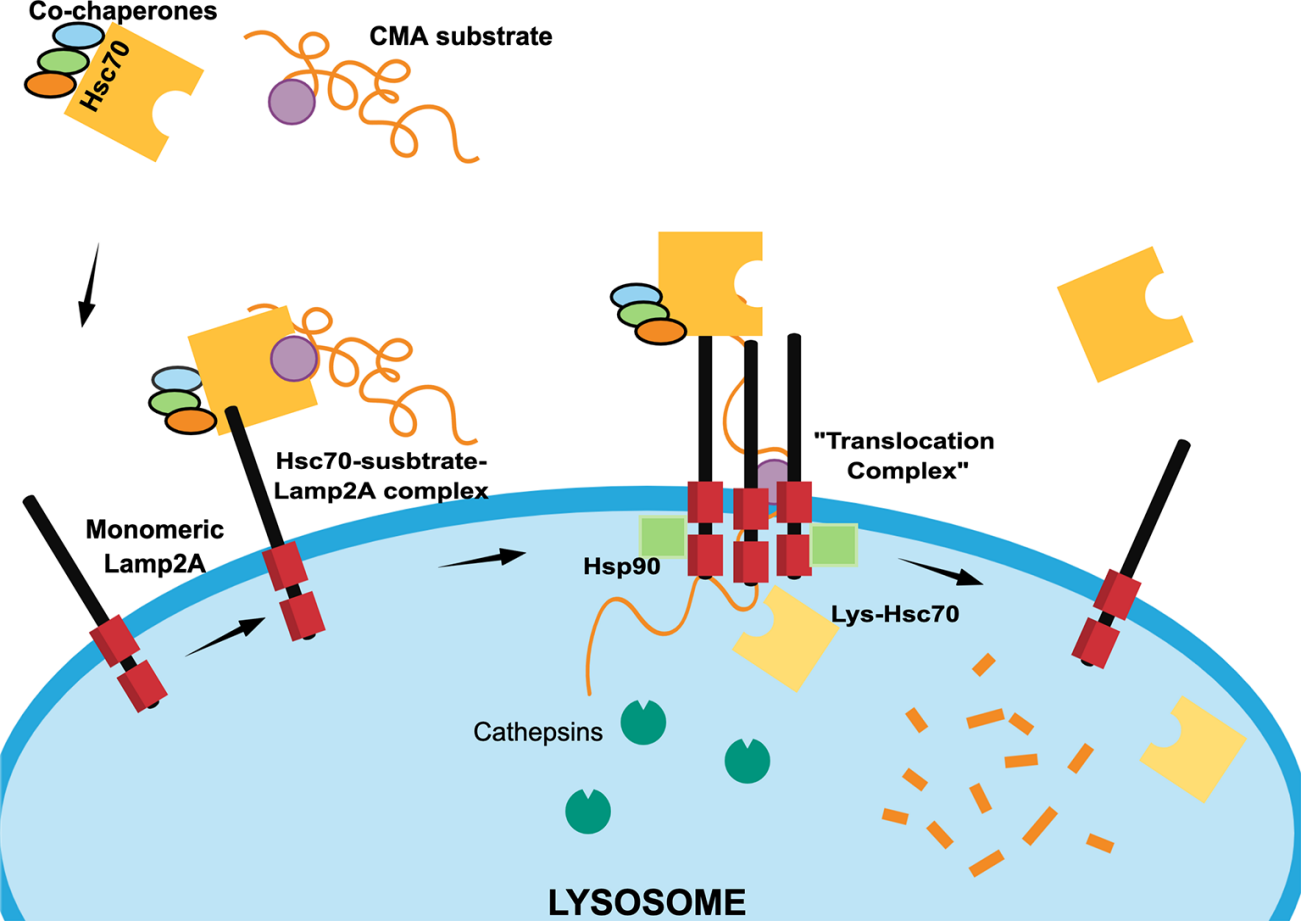
Scheme of different chaperone-mediated autophagy (CMA) steps. In the cytosol, Hsc70 heat shock protein recognizes CMA protein substrates containing KFERQ-like motifs (purple circle) with the assistance of co-chaperones Hip, Hop and Hsp40 (light blue, green and orange ovals) to form a “Hsc70-substrate complex”. The Hsc70-substrate complex interacts with monomeric Lamp2A at the lysosomal membrane to induce the formation of a “translocation complex” through Lamp2A oligomerization, which is regulated at the outer lysosome membrane by the Glial Fibrillary Acidic Protein (GFAP), Protein Phosphatase 1 (PHLPP1), and Elongation Factor 1α (EF1α) proteins (not shown). Once the “*translocation complex*” is formed, the CMA substrate protein unfolds to enters at the lysosomal lumen thanks to the assistance luminal Hsc70 (lys-Hsc70) and Hsp90 (green squares). Once, the CMA substrate protein is degraded by cathepsins and other proteases, the “*translocation complex*” dissociates, Hsc70 is recycled and Lamp2A returns to a monomeric state.

Lamp2A is found in a monomeric state at the lysosomal surface and, when the substrate-chaperone-Lamp2A complex is formed, Lamp2A initiates a multimerization process (15). This process is assisted in the cytosolic side by positive regulators including Glial Fibrillary Acidic Protein *(*GFAP), PH domain Leucine-rich repeat-containing Protein Phosphatase 1 (PHLPP1), and Elongation Factor 1α (EF1α) (16) that regulate and stabilize the multimer. Once stabilized, the Lamp2A multimer serves as a “*translocation complex*” by which the substrate is unfolded and translocated into the lysosomal lumen. Inside the lysosome, the presence of a luminal chaperone lys-Hsc70 is also required to complete the substrate internalization (14, 17) (**Figure 2**).

## CMA Activity in Different Cancer Types

Altered CMA activity has been reported in different physiopathological processes such as metabolic alterations, neurodegeneration and aging (12), where the abnormal degradation of the substrates are contributing to the progression of the cellular damage. Changes in CMA activity also has been addressed for different types of cancer. For example, through immunostaining and fluorescent reporter proteins, studies in several cancer cell lines such as lung (A549, H460), breast (MCF7), liver (HUH7), epithelial (Saos 2 osteosarcoma), stomach, colon, uterus, and ovary, have concluded that CMA activity is constitutively upregulated in cancer cells (18). Interestingly, such up-regulation seems to be independent of the macroautophagy status, suggesting a specific role of CMA in these cancer types (19–21). Taking into account the different cancer cell lines studied, it was possible to suggest that CMA is found to be upregulated 2.8-fold with respect to non-oncogenic control cells (18). In this sense, it becomes important to understand the role that CMA up-regulation can be playing in the development of cancer pathology. The most accepted theory is that CMA contributes to maintain the malignant condition by preventing the entry of the cells into an apoptotic process and protecting them from stress (13, 22). Taking into account these two aspects, and the observations made by some authors (18), CMA up-regulation could be considered as a pro-survival cancer cell factor and thus its activity inhibition could be the target for therapeutics. However, the latter is not a general rule since recent studies also indicate that CMA has an anti-oncogenic role by limiting malignant transformation in some types of cancer (13, 23).

On the other hand, a very recent review describing the role of CMA in regulating the cell cycle by keeping the balance between negative and positive cell cycle regulators suggests that a CMA impairment could lead to tumor progression and cell malignant transformation (24). Further, an additional report has recently showed that CMA is able to drive the balance between stem cell proliferation and cell differentiation, two cellular properties that are closely related with cancer (25).

Thus, up to date, the exact role of CMA in cancer is uncertain and it will depend on each cancer type. Probably, a way to determine how CMA is participating in the progression of cancer is to correctly interpret the significance that alterations of specific CMA substrates or CMA components might have in the different cancer types.

## CMA Substrates in Cancer: Different Perspectives to Approach the Problem

Different confirmed or potential CMA substrates in different cancer types accumulate despite the fact that CMA activity is increased (26). Some of these CMA substrates are pro-oncogenic and are protected from CMA degradation by different reasons. Thus, cancer progression not always will depend on the status of CMA activity but also on the altered CMA-dependent degradation of the anti- and/or pro-oncogenic protein substrates (**Table 1**).

**Table 1:** Protein acting as pro-oncogenic or anti-oncogenic factors, validated or not as chaperone-mediated autophagy (CMA) substrate and its role in different cancer types.

Cancer type	Altered protein in cancer	Normal function	Activity in cancer	KFERQ motif	Protected from degradation	Reference
Breast, Ovarian, cancers Fibrosarcome	**Mutant P53**	Apoptotic pathway	Promoter	Non canonical Proved CMA substrate	Yes	Vakifahmetoglu-norberg et al. (27)
Breast, Prostate cancer	**HSD17B4** 17b- hydroxysteroid dehydrogenase type 4	Fatty acid β-oxidation and steroid metabolism	Promoter	Non canonical Not proved CMA substrate	Yes	Huang et al. (28)
Acute Myeloid leukemia erythroleukemia	**HKII** Hexokinase II	Glucose metabolism	Promoter	Canonical Proved CMA susbtrate	Yes	Xia et al. (29)
Breast cancer	**MORC2** MORC family CW-type zinc finger 2	Lipogenesis and adipogenesis	Promoter	Canonical Proved CMA susbtrate	Yes	Yang et al. (30)
Pancreatic cancer	**IGF-IR** insulin-like growth factor 1 receptor	Proliferation and cell growth	Promoter	Non canonical Not proved	Yes	Xue et al. (31)
Hepatocellular carcinoma Cervical cancer	**HIF 1α** Hypoxia-inducible factor 1α	Hypoxia response Cell cycle regulator	Promoter	Non canonical Proved CMA substrate	No	Hubbi et al. (32)
Gastric cancer	**RND3** Rho Family GTPase 3	Anti-proliferative Cell cycle regulator	Suppressor	Canonical Proved CMA substrate	No	Zhou et al. (33)
Breast cancer	**ATG-5** Autophagy-related gene 5	Autophagy	Suppressor	Non canonical	No	Han et al. (34)
Colorectal	**SNX10/P21** Sorting nexin 10/protein 21	endosome/lysosome homeostasis/cell cycle regulator	Suppressor	Non canonical Proved CMA substrate	No	Zhang et al. (35)

One of the most known CMA substrate that is protected from CMA degradation is P53, an important tumor suppressor protein encoded by the *tp53* gene. Whereas wild type P53 protein is a poor CMA substrate, missense mutations in the *tp53* gene leads to a mutant form of P53 which favors its degradation by CMA in the cytosol. Mutant P53 accumulates in the nucleus exerting its pro-oncogenic role and protecting itself from degradation, but under conditions of stress and autophagy inhibition, mutant P53 translocate to the cytosol and interacts with Hsc70 followed by CMA degradation (27). The latter example suggests that, in particular cases, a strategy to counteract the cancer progression can be to expose the mutant pro-oncogenic substrate to be degraded by CMA.

In addition, there is evidence indicating that alteration of CMA substrates through post-translational modifications such as acetylation or phosphorylation can also represent an appropriate alternative to reduce cell malignance. For example, in prostate cancer cells, the up-regulation of HSD17B4, a protein involved in fatty acid oxidation, is correlated with tumor progression (28). HSD17B4 acetylation enhances its interaction with Hsc70 and Lamp2A, allowing its degradation through CMA. However, it was shown that deacetylation processes that are predominant in prostate cancer (28) protects HSD17B4 from CMA degradation and contributes with cancer progression. Phosphorylation is another modification that protects CMA substrates from degradation. Hexokinase II (HKII) is a key enzyme involved in glucose metabolism and is highly expressed in cancer cells (36). Interestingly, in breast cancer, HKII phosphorylation protects the protein from its degradation through CMA, promoting proliferation, migration and tumor growth (37). However, when using a specific HKII phosphorylation inhibitor, tumor growth is reduced (37). In addition, in gastric cancer cells, some members of the Rho family like RND3, are maintained in the cytosol by specific phosphorylation events, which in turn allow their interaction with CMA components followed by lysosomal degradation (38). A similar example is the novel oncogene MORC2 (MORC family CW-type zinc finger 2), that has been found at high levels in breast cancer promoting metastatic progression. This protein is protected from CMA degradation by a phosphorylation that prevents its interaction with Hsc70 (39). Thus, the inhibition of MORC2 phosphorylation to allow its degradation through CMA could be a good approach to reduce the cell malignance.

Finally, to expose the KFERQ-like motif can be also an option to drive the degradation of a pro-oncogenic CMA substrate. For example, it was shown that inhibition of macroautophagy in ovarian cancer cells in the absence of glucose exposes the HKII KFERQ-motif and allows its recognition by Hsc70, directing its degradation through CMA (29). A similar situation occurs with the IGF-IR (insulin-like growth factor-1), a family member of insulin receptors that is enhanced in different cancer types (39). In pancreatic cancer, IGF-IR acts as pro-oncogene and remains protected from CMA degradation by its interaction with chaperone Hsp90. If this interaction is disrupted by specific Hsp90 inhibitors, IGF-IR is exposed for Hsc70 recognition and is degraded through CMA (31).

There are cases where the direct CMA up-regulation can effectively drive the degradation of a particular CMA substrate to prevent cancer progression. Hypoxia-inducible factor 1 (HIF-1), is a transcription factor composed by two subunits, HIF-1α and HIF-1β and is required as an adaptive response to low oxygen conditions and necessary for tumor progression (40, 41). Hsc70 and Lamp2A knock-down led to an increase of HIF-1α protein level in cancer cell lines such as HeLa and Hep3b (32). On the other hand, high expression levels of Hsc70 and Lamp2A in the same cell lines were associated with reduced protein of HIF1α  (32). Thus, in this case, the direct enhancement of CMA activity can promote the degradation of this pro-oncogenic protein. Another example is the case of RND3 (Rho Family GTPase 3), an anti-proliferative protein that has been previously confirmed as CMA substrate (33). High expression of Lamp2A was correlated with reduced RND3 protein levels in cancer gastric cells, and the silencing of Lamp2A was associated with increased RND3 protein levels and inhibited cell proliferation (33). Therefore, in this particular case, inhibition of CMA activity could be considered as a therapeutic alternative to implement. Autophagy-related gene 5 (ATG5) acts as a tumor suppressor protein and its absence causes tumor generation in different tissues (42), including breast. Also, in breast cancer, Lamp2A protein is abnormally upregulated (43) and plays an important role in cell survival and metastasis (34). Interestingly, in a breast cancer model it was shown that Lamp2A down-regulation induces ATG5 protein levels increase and inhibited cancer cell growth and metastasis (34), suggesting that an inhibition of CMA activity could be an option to reverse the malignant phenotype dependent on ATG5 protein alterations.

Finally, in colorectal cancer cells, Lamp2A expression levels are upregulated as a consequence of the lower protein levels of sorting nexin 10 (SNX10) (35). The latter leads to increased CMA activity and exacerbated degradation of the tumor suppressor protein P21, allowing the proliferation of colon cancer cells (35). Thus, in this particular case, overexpression of SNX10 protein results to be a good alternative to reduce CMA activity and allows the accumulation an anti-oncogenic action of P21.

## Lamp2A and Hsc70 in Cancer Beyond CMA

As mentioned above, Lamp2A and Hsc70 are two proteins whose role is essential for the CMA pathway, in particular Lamp2A that has been reported to be the limiting step of the pathway (44). Up to date, many evidences have stablished that in addition to evaluate the levels of Lamp2A and Hsc70, additional requirements and functional assays are necessary to determine whether changes in CMA activity occurs in a determined cellular state. In particular, immunoblots of Lamp2A and Hsc70 in total cell lysates or tissue homogenates are less valuable to determine CMA activity as their changes should be confirmed principally in lysosomes (17, 44, 45). Sometimes, in various cell types, a useful indirect indication of CMA activation can be to evaluate the distribution of hsc70-positive lysosomes toward the perinuclear region (46). Additionally, in total cell lysates or tissue homogenates, and particularly when using isolated lysosomes from cells or tissues, changes in the levels of CMA substrates is a good indication of alteration in CMA activity (46). Finally, applying some functional assays such as the use of photoconvertible CMA reporters, the assessment of intracellular protein degradation or the *in vitro* analysis of CMA using isolated lysosomes, are all very well accepted approaches to confirm changes in CMA activity (44).

Thus, considering all the exposed above, additional considerations should be taken before to conclude that alterations in Lamp2A and Hsc70 levels are directly correlated with changes in CMA activity, and the altered expression of these proteins might have a different significance for the cancerous cell. In fact, these proteins would fulfil additional functions in the cell, in particular Hsc70 (47, 48). Next, we discuss about some evidences reporting alterations in Lamp2A and Hsc70 expression but where changes in CMA activity was not clearly confirmed, suggesting that the alteration in Lamp2A or Hsc70 may have a different role in the cancerous cells.

### Lamp2A Protein

By alternative splicing, the *lamp2* gene can generate three different isoform proteins; Lamp2A, Lamp2B, and Lamp2C, where only Lamp2A has been exclusively linked to CMA activity (45). In patients with breast cancer, Lamp2A overexpression was detected in all samples containing cancerous tissues and was correlated to cancer cell survival (43). In addition, Lamp2A has also been used as a marker to measure the lysosomal content in breast carcinomas and was correlated with increased activity of the transcription factor EB (TFEB). Interestingly, in another study in breast cancer showed that 30% of the cancerous samples displayed Lamp2A overexpression (49). Although the exact role of Lamp2A in breast cancer progression should be further clarified, it is clear that this protein is relevant for cancerous cell survival demonstrated by the inhibition of cell migration and invasion in silenced cells (34). In addition, when Lamp-2A was experimentally up-regulated an increase in cell growth was observed (34). In addition to breast cancer, other cancer types have reported altered Lamp2A expression without an evident analysis on the changes in CMA activity. For example, reduced Lamp2A expression was required to avoid apoptosis and promote cell proliferation in hepatocellular carcinoma xenograft growth (50). Further, in several gastric cancer cells, Lamp2A overexpression was proposed as a good marker for early cancer prediction in precancerous lesions (33). Additionally, in Non-Small-Cells Lung Cancer (NSCLC), an increase in Lamp2A expression was observed compared to healthy tissues (21). Further, in Tumor-associated macrophages (TAM), it was demonstrated that supernatants from tumor cells, which contains growth and inflammatory factors, induce Lamp2A up-regulation in TAM and allow its activation, favoring the tumor growth maintenance (51). Lamp2A silencing by shRNA or CRISPR-Cas9 was correlated with an increase in PRDX1 (peroxiredoxin 1) and CRTC1 (CREB-regulated transcription coactivator 1), two factors necessary to promote macrophage pro-tumorigenic activation. Although CMA activity was not evaluated, the increase in PRDX1 and CRTC1 after Lamp2A down-regulation suggest that in this case a CMA impairment in TAM can be related with tumor growth (51). Further, Lamp2A down-regulation reverted the tumorigenic features of TAM cells (51). Finally, an increased expression of Lamp2A has been found in tissues from patients with colorectal cancer Stage II and III, and the Lamp2A silencing in the colorectal cell line CT26 induced apoptosis and inhibited cell proliferation (19). Taken all together, Lamp2A overexpression is mostly associated to cancer progression independent of observable or reported changes in CMA activity.

### Hsc70 Protein

**Figure 3:**
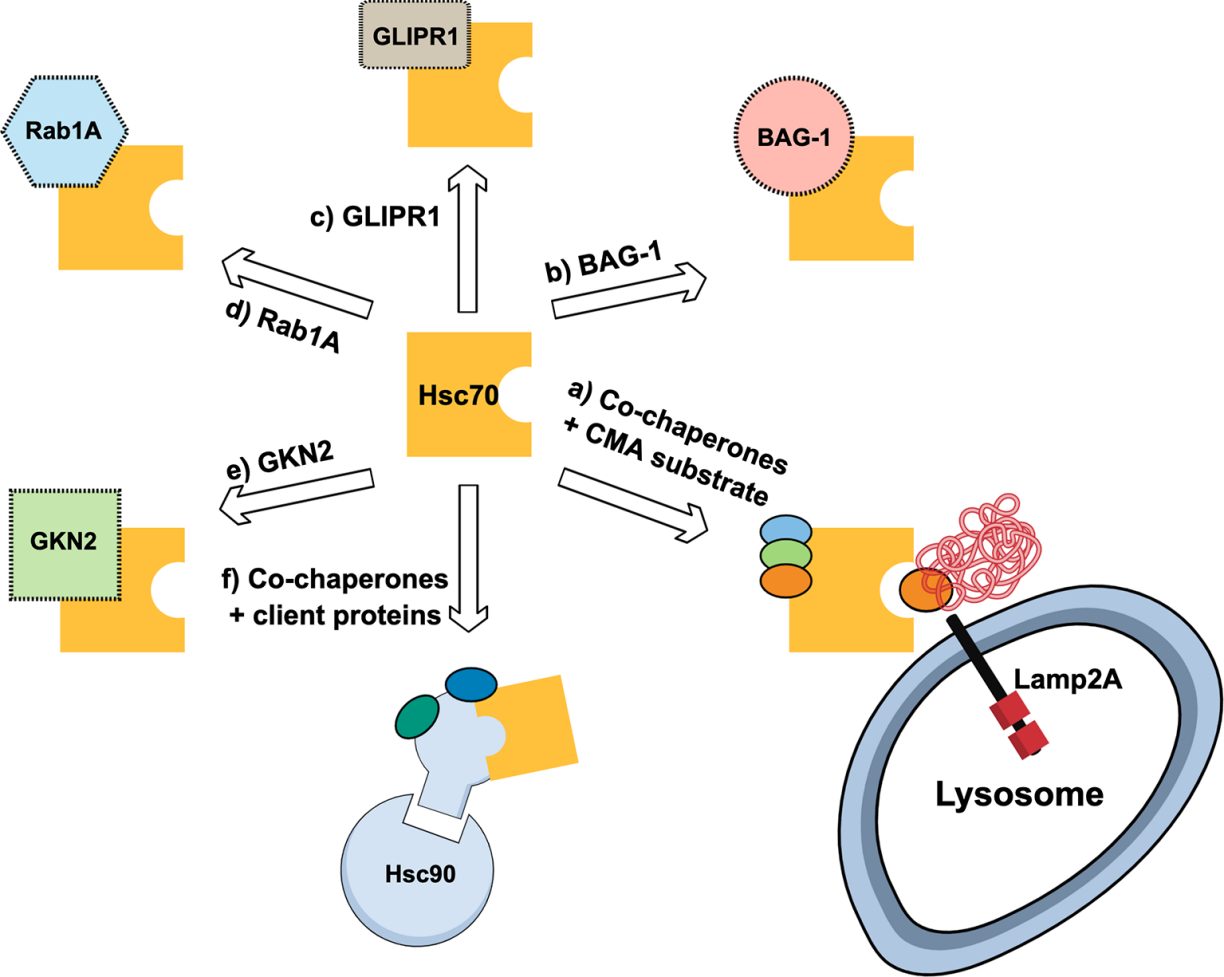
Schematic representation of multiple routes where Hsc70 is associated with cancer. **(A)** shows Hsc70 forming complex with additional chaperones and recognizes chaperone-mediated autophagy (CMA) substrates for lysosomal degradation by Lamp2A-mediated internalization; **(B)** shows Hsc70 interaction with BAG-1, a co-chaperone with anti-apoptotic activity; **(C)** Hsc70 interacts with Glioma Pathogenesis-related Protein 1 (GLIPR1) related to glioma and glioblastoma multiforme; **(D)** Rab1A, a protein related with cancer cell survival, is protected from degradation by interacting with Hsc70, despite Rab1A has canonical KFERQ motif; **(E)** Hsc70 complexes with Gastrokine-2 (GKN2), a secretory protein associated with gastric cancer; **(F)** Hsc70 acting as cochaperone to deliver “client proteins” to Hsc90 machinery.

Hsc70 is a cytoplasmic protein that belongs to the Hsp70 multigene family. An important aspect of Hsc70 is that its expression is constitutive and independent to a heat shock response (52). This chaperone has been described to interact with several cancer-related proteins (47) and thus its role in cancer includes its interaction with related and non-related CMA proteins, and with its altered expression levels in cancerous cells or tissue (**Figure 3**). Hsc70 is part of the co-chaperones required for the delivery of “client proteins” to Hsp90 machinery (53). Further, the development of Hsp90 inhibitors has been the focus of many studies due to the implications of this protein in cancer progression (54). However, the inhibition of Hsp90 might induce Hsc70 overexpression which counteract the antitumorigenic effect of the Hsp90 inhibitor (55). Thus, the silencing of Hsc70 in different cancer cell lines, together with the use of Hsp90 inhibitors, has been used to cause cell cycle arrest and increase in tumoral cell apoptosis in response to Hsp90 inhibitors (55). In line with this, it was observed that the use of a Hsp90 inhibitor in B-lineage acute lymphoblastic leukemia cells allowed the induction of Hsc70, cathepsin D inhibition and apoptosis induction of the cancerous cells (56). In addition, BAG-1 a multifunctional protein related to diverse cancer types (57), also interacts with the Hsc70 amino-terminal ATP-binding domain (58). When using peptides resembling the BAG domain in breast cancer cells, the interaction between BAG-1 and Hsc70 is prevented and cell growth is arrested (59). Moreover, the interaction of Hsc70 with glioma pathogenesis-related protein (GLIPR1) was associated with oncogenic signaling pathway by suppressing the transcription of AURKA and TPX2 by SP1 and c-Myb destabilization (60). Furthermore, it has been observed that Rab1A overexpression is vital for the maintenance of cancer cell physiology under stress conditions. With this regard, the ubiquitination and protein degradation of Rab1A was shown to be protected due to its interaction with Hsc70 (61). Thus, in some cancer types, Hsc70 expression can favor cancer progression by, for example, replacing the function of a related protein (e.g., Hsp90) or by protecting a pro-oncogenic factor from its degradation.

However, in other cancer types, Hsc70 can be beneficial by promoting cancer arrest. For example, the overexpression of GKN2 protein sensitized gastric cancer cells to apoptosis under oxidative stress inducers. However, the effect of GKN2 to induce apoptosis was dependent on its interaction with Hsc70 (62). Also, Hsc70 has also been described as an important player in the mechanism regulating the suppression of colorectal cancer metastasis by its interaction with the amino-terminal enhancer of split protein (Aes) (63). Finally, it was shown that colon cancer tissues displayed increased expression and protein complexes of Hsp70, Hsp90 and HOP compared with control tissues, suggesting an association between cancer progression and complexes formation (64).

Additionally, some works have associated the variations in Hsc70 expression as part of cell-to-cell communication, or as a biomarker of cancer progression. In healthy cells, Hsc70 remains intracellular but in the lymphoblastic cell line K562 and in human breast cancer cells, Hsc70 is secreted to the media and considered as a new signal of growth arrest when cells are confluent (65, 66). Hsc70 has also been proposed as a biomarker for endometrial carcinoma due to its up-regulation in cancer tissues (67). Further, the progression of human brain glioma cells and tumor grade was associated with Hsc70 overexpression. In particular, the migration and invasion of these cells was inhibited by a Hsc70 downregulation (68).

Altogether, these evidences further indicate that, depending on each cancer nature, the altered Hsc70 expression might have additional or different effects on cancer progression, rather than modified CMA activity.

## Concluding Remarks

Most of the analyzed evidence, report an up-regulation in Lamp2A and Hsc70 CMA components in the different cancer cell types studied. When the up-regulation of these CMA components were associated with an increase in CMA activity, it was mostly connected with a protective mechanism for stress microenvironment and lack of nutrients, contributing to tumor growth and cancer progression. The latter suggests that inhibition of CMA activity could be a target for therapeutic approaches to increase the sensitivity of tumor cells to stress, promote cell death and decreased tumor growth. However, few evidences also show that CMA activity would contribute to cancer arrest and thus to inhibit or enhance the CMA activity probably will depend on each specific cancer type.

In addition, whereas several proteins involved in the progression of different cancer types have been experimentally well-defined as CMA substrates, others, although having putative KFERQ-like motifs or indirectly associated to changes in CMA activity, have not fully been defined as CMA substrates (**Table 1**). With this regard, although the enhancement or inhibition of CMA can be the objective to reverse the cancer phenotype, also the efforts could be made in modifying protein substrates (e.g., by altering posttranslational modifications, cellular conditions or protein-protein interactions) to promote or inhibit its degradation through CMA, depending on the particular case.

Many evidences have stablished that, in addition to evaluate the levels of Lamp2A and Hsc70, further assays are necessary to determine whether changes in CMA activity occurs in a determined cellular state. According to this, some reports have showed alterations in Lamp2A and Hsc70, without evident assayed changes in CMA activity. The latter leaves the open question about if the changes in Lamp2A or Hsc70 expression in these particular cases can be fulfilling an additional role in carcinogenesis beyond modifying the CMA activity.

In conclusion, depending on each specific cancer type, it will be important to experimentally determine whether detectable alterations in Lamp2A and Hsc70 expression have a direct impact on CMA activity. If it is the case or not, then the question should be whether targeting CMA activity is an option to counteract cancer progression, or whether the attention of the strategy must be focused on Lamp2A or Hsc70, or some particular CMA protein substrate.

## Author Contributions

JR and AS analyzed the bibliography, wrote the manuscript, and made figures and table. AA made revisions and editing. MB analyzed the bibliography, contributed with writing, and made substantial revisions and editing of the manuscript. All authors contributed to the article and approved the submitted version.

## Funding

This work was funded by CONICYT PIA ACT172066 to MB.

## Conflict of Interest

The authors declare that the research was conducted in the absence of any commercial or financial relationships that could be construed as a potential conflict of interest.

## Abbreviations

ATG-5, Autophagy-related gene 5; CMA, Chaperone-Mediated Autophagy; CRTC1, CREB-regulated transcription coactivator 1; EF1, Elongation Factor 1; GFAP, Glial Fibrillary acidic protein; GKN2, Gastrokine 2; GLPR1, Glioma pathogenesis-related protein 1; HIF-1, Hypoxia-inducible factor 1; HKII, Hexokinase II; Hsc70, Heat Shock cognate 70 KDa; HSD17B4, Hydroxysteroid 17-Beta Dehydrogenase 4; Hsp90, Heat Shock protein 90 Kda; IGF-IR, Insulin-like growth factor-1; Lamp2A, Lysosomal-associated membrane protein type 2A; Lys-HSc70, Lysosomal Heat Shock Cognate 70 KDa; MORC2, MORC family CW-type zinc finger 2; NSCLC, Non-Small-Cells Lung Cancer; PHLPP1, PH domain leucine-rich repeat-containing protein phosphatase 1; PRDX1, Peroxiredoxin 1; RND3, Rho Family GTPase 3; SNX-10, Sorting Nexin 10; TAM, Tumor associated macrophages; TFEB, Transcription Factor EB.
